# Supplementary material for: Checkpoint-Dependent Sensitivities to Nucleoside Analogues Uncover Specific Patterns of Genomic Instability
Source: Curr Issues Mol Biol. 2025 Sep 12;47(9):756. doi: 10.3390/cimb47090756 (PMC12468036; doi:10.3390/cimb47090756)
Supplement: Supplementary file 1 [file cimb-47-00756-s001.zip › cimb-3786261-supplementary.pdf]

SUPPLEMENTARTY DATA FILE
for

Checkpoint-dependent sensitivities to nucleoside analogues uncover specific patterns of genomic instability

Zainab Kagalwala[1,2,‡], Mohammed Ayan Chhipa[1,3,‡], Zohreh Kianfard[1,3], Essam Karam [1,3], Sirasie P Magalage[1,3], and Sarah A Sabatinos[1,3,*]

Contents:

S1: Supplementary Figure S1. Insensitivity to FUdR increases above the IC50 dose of BrdU.

S2: Supplementary Figure S2. IC50 dose curves for hydroxyurea (HU).

Supplementary Figure S1:

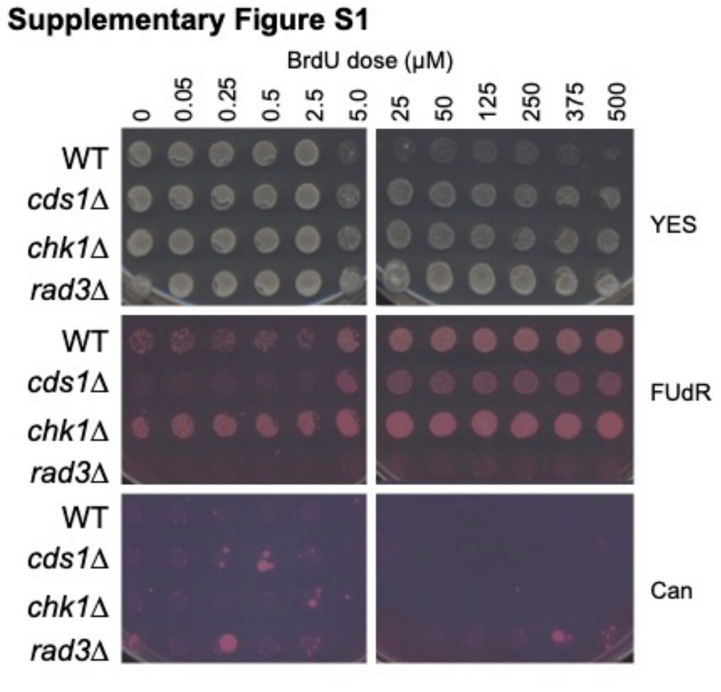

Supplementary Figure S1. Insensitivity to FUdR increases above the IC50 dose of BrdU. Shown on top are the YES plates after 48 h exposure to varying doses of BrdU in PMG medium with supplements. Concurrently, treated cells were also pinned onto FUdR (middle) to assess HSV-TK function. Cells were also pinned onto PMG-HULA medium containing 70 μg/mL canavanine sulfate [80]. Canavanine (Can) can be used to detect forward mutation in the canavanine sensitivity pathway. Can mutants are large colonies and are more common in *cds1*Δ, *chk1*Δ and *rad3*Δ up to the IC50 dose. Phloxine B was added to FUdR and Can plates to assess cell health; darker pink staining means that cells are less able to excrete the dye and the spots are darker pink.

Supplementary Figure S2

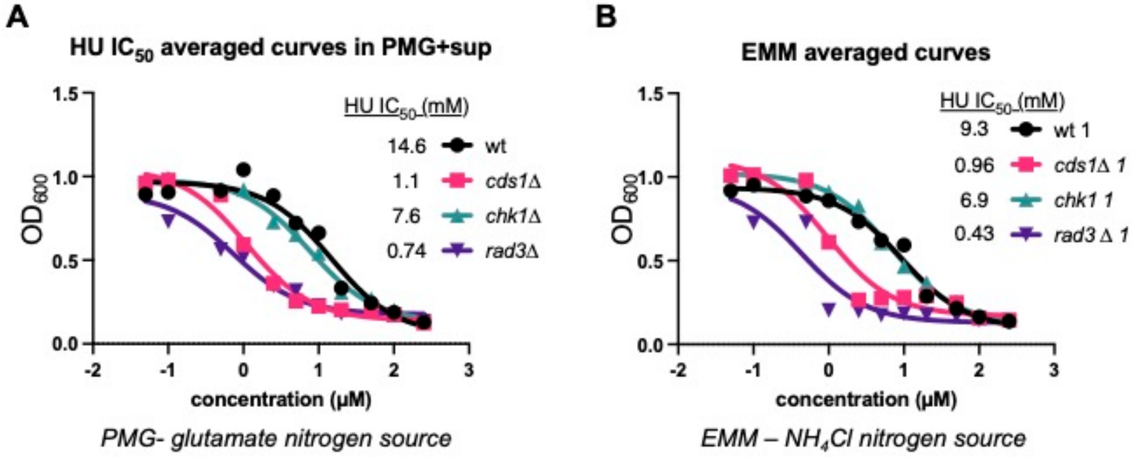

Supplementary Figure S2. Supplementary Figure S2. IC50 dose curves for hydroxyurea (HU). Strains (wild type, *cds1*Δ*, chk1*Δ*, rad3*Δ*)* were exposed to an 11-dose IC50 curve of HU using OD600 in either PMG or EMM medium. Shown are aggregated data from 2 experimental replicates, with the calculated IC50 value in mM. PMG medium (S2A) contains glutamate as a nitrogen source. EMM medium (S2B) contains ammonium chloride as the nitrogen source. Both media were supplemented at 225 mg/L with his, ura, leu, ade.

HU inhibits ribonucleotide reductase, which decreases dNTP levels and causes DNA replication arrest; this causes a *rad3*Δ- and *cds1*Δ-dependent checkpoint response. The *rad3*Δ and *cds1*Δ cells are sensitive to lower doses of HU than wild type or *chk1*Δ. These data contrast with the IC50 curves/values calculated for the various nucleoside analogues in Figures 2 and 3. Because nucleoside analogues affect multiple aspects of DNA replication stability (RNR activity, polymerase activity, mRNA transcription), the spectrum of IC50 result(s) may not be cleanly “HU-like” in different analogues and checkpoint mutant variables.
